# Smoking, white blood cell counts, and TNF system activity in Japanese male subjects with normal glucose tolerance

**DOI:** 10.1186/1617-9625-9-12

**Published:** 2011-11-25

**Authors:** Naoya Watanabe, Mitsuo Fukushima, Ataru Taniguchi, Takahide Okumura, Yoshio Nomura, Fusanori Nishimura, Sae Aoyama, Daisuke Yabe, Yoshio Izumi, Ryoichi Ohtsubo, Yoshikatsu Nakai, Shoichiro Nagasaka

**Affiliations:** 1 Health Care and Promotion Center, Yodogawa Christian Hospital, 2-9-26, Awaji, Higashiyodogawa-ku, Osaka, 533-0032, Japan; 2 Division of Clinical Nutrition and Internal Medicine, Okayama Prefectural University, 111, Kuboki, Soja-city, Okayama, 719-1197, Japan; 3 Division of Diabetes and Endocrinology, Kyoto Preventive Medical Center, 28, Nishinokyo, Samaryocho, Nakagyo-ku, Kyoto, 604-8091, Japan; 4 Department of Pharmacy, Saiseikai-Nakatsu Hospital, 2-10-39, Shibata, Kita-ku, Osaka, 530-0012, Japan; 5 Department of Oral Health, Kobe Tokiwa Junior College, 2-6-2, Otani-cho, Nagata-ku, Kobe, 653-0838, Japan; 6 Department of Dental Science for Health Promotion, Hiroshima University Graduate School of Biomedical Sciences, 1-2-3, Kasumi, Minami-ku, Hiroshima, 734-8551, Japan; 7 Division of Diabetes, Clinical Nutrition and Endocrinology, Kansai Electric Power Hospital, 2-1-7, Fukushima, Fukushima-ku, Osaka, 553-0003, Japan; 8 Department of Internal Medicine, Kyoto Postal Services Agency Hospital, 109,Nishirokkakucho, Rokkaku-street, Shinmachi, Nakagyo-ku, Kyoto, 604-8798, Japan; 9 Kyoto Institute of Health Science, Karasumaoike-Higashi, Nakagyo-ku, Kyoto, 604-0845, Japan; 10 Division of Endocrinology and Metabolism, Jichi Medical University, 3311-1, Yakushiji, Shimono-city, Tochigi, 329-0498, Japan

## Abstract

**Background:**

Cigarette smokers have increased white blood cell (WBC) counts and the activation of tumor necrosis factor (TNF). The effect of smoking on WBC counts and TNF system activity, however, has not been separately investigated yet.

**Subjects and Methods:**

One hundred and forty-two Japanese male subjects with normal glucose tolerance were recruited. They were stratified into two groups based on the questionnaire for smoking: one with current smokers (n = 48) and the other with current non-smokers (n = 94). Whereas no significant differences were observed in age, BMI, high molecular weight (HMW) adiponectin, and TNF-α between the two groups, current smokers had significantly higher soluble TNF receptor 1 (sTNF-R1) (1203 ± 30 vs. 1116 ± 21 pg/ml, *p *= 0.010) and increased WBC counts (7165 ± 242 vs. 5590 ± 163/μl, *p *< 0.001) and lower HDL cholesterol (55 ± 2 vs. 60 ± 1 mg/dl, *p *= 0.031) as compared to current non-smokers. Next, we classified 48 current smokers into two subpopulations: one with heavy smoking (Brinkman index ≥ 600) and the other with light smoking (Brinkman index < 600).

**Results:**

Whereas no significant difference was observed in age, BMI, HMW adiponectin, WBC counts and TNF-α, sTNF-R1 and sTNF-R2 were significantly higher in heavy smoking group (1307 ± 44 vs. 1099 ± 30 pg/ml, *p *< 0.001; 2166 ± 86 vs. 827 ± 62 pg/ml, *p *= 0.005) than in light smoking group, whose sTNF-R1 and sTNF-R2 were similar to non-smokers (sTNF-R1: 1116 ± 15 pg/ml, *p *= 0.718, sTNF-R2; 1901 ± 32 pg/ml, *p *= 0.437). In contrast, WBC counts were significantly increased in heavy (7500 ± 324/μl, *p *< 0.001) or light (6829 ± 352/μl, *p *= 0.001) smoking group as compared to non-smokers (5590 ± 178/μl). There was no significant difference in WBC counts between heavy and light smoking group (*p *= 0.158).

**Conclusion:**

We can hypothesize that light smoking is associated with an increase in WBC counts, while heavy smoking is responsible for TNF activation in Japanese male subjects with normal glucose tolerance.

## Background

It is well recognized that smoking is one of the most important factors contributing to the evolution of atherosclerosis and chronic obstructive pulmonary disease in humans [1,2]. It is well established that current smokers are characterized by increased white blood cell (WBC) counts and increased tumor necrosis factor-α(TNF-α) [3,4]. However, the effects of smoking on the increase in WBC counts and TNF-α have not been separately investigated yet. In this regard, a major problem is that compared with circulating TNF-α, soluble TNF receptor (soluble TNF receptor 1 (sTNF-R1) and soluble TNF receptor 2 (sTNF-R2)) levels remain elevated for longer periods of time and are of more value for monitoring TNF system activities. Another problem is that the degree of glucose tolerance or of body mass index per se is considered to affect WBC counts and TNF system activity in man. To overcome these difficulties, we recruited the subjects with normal glucose tolerance whose BMI is less than 29.0 kg/m^2 ^and measured soluble TNF receptors along with TNF-α.

## Subjects and Methods

### Participants and Setting

A total of 142 Japanese male subjects with normal glucose tolerance who visited Yodogawa Christian Hospital for the medical checkup were enrolled for the present study after the ethics committee of Yodogawa Christian Hospital approved the protocol and informed written consents were obtained from every participant. Body mass index in these subjects was 23.2 ± 0.2 kg/m^2 ^(mean ± SEM). Exclusion criteria were cancer, ischemic heart disease, cerebral stroke, renal disease, hepatic disease, asthma, or any inflammatory disease such as rheumatoid arthritis or inflammatory bowel disease. Six (4%) of the 142 subjects were treated with antihypertensive medications. Twelve (8%) of the 142 subjects were treated with lipid lowering agents. No subjects have received any medications known to alter glucose tolerance. Before the study, they did not use anti-inflammatory medication. All subjects had ingested at least 150 g of carbohydrate for the 3 consecutive days preceding the study. They did not consume alcohol or perform heavy exercise for at least one week before the study.

### Definitions of Active Smoking

Smokers and non-smokers were determined based on the questionnaire for smoking. Smokers were further classified into two subgroups based on the value of Brinkman index; one with heavy smoking (Brinkman index ≧ 600) and the other with light smoking (Brinkman index < 600). Brinkman index was calculated as the product of the number of tobacco smoked and inhaled year [5]. Miyatake et al. [6] recently demonstrated that the subjects with a Brinkman index ≧ 600 are at increased risk for metabolic syndrome as compared to those with a Brinkman index less than 600 in Japanese men.

### Blood Analysis

Blood was drawn from the antecubital vein in the morning after a 12-hour fast. Plasma glucose, triglyceride, total cholesterol, HDL cholesterol, LDL cholesterol, white blood cell counts, serum creatinine, and hemoglobin A1c were measured. Hemoglobin A1c (HbA1c) was shown in NGSP values as recommended by the Japan Diabetes Society [7,8]. Along with TNF-α, soluble TNF receptors (sTNF-R1, sTNF-R2) were measured in the present study. Serum TNF-α concentrations were measured by enzyme immunoassay kit (Quantikine HS Human TNF-α immunoassay kit, R&D systems. Inc, Minneapolis, MN, USA) and serum concentrations of sTNF-R1 and sTNF-R2 were measured by enzyme-linked immunosorbent assay (ELISA) (BIOTRAK, Amersham Life Sciences, Uppsala, Sweden), as described previously [9-12]. The limits of sensitivity for TNF-α, sTNF-R1 and sTNF-R2 were 0.5 pg/ml, 25 pg/ml and 50 pg/ml, respectively. The intra-assay and interassay coefficients of variation were less than 8% for TNF-α, sTNF-R1 and sTNF-R2. Insulin and high molecular weight adiponectin (HMW adiponectin) were measured as described previously [11,13]. Samples for TNF, insulin, and HMW adiponectin were prepared, frozen, and stored at -70°C until the assay. The estimate of insulin resistance by HOMA (HOMA-IR) was calculated with the formula: fasting serum insulin (μU/ml) × fasting glucose (mmol/l)/22.5 [14]. We previously demonstrated that HOMA-IR is strongly correlated with minimal model derived insulin sensitivity in Japanese subjects with varying degrees of glucose tolerance [15]. Body fat percentage was measured using bioelectrical impedence method.

### Statistical Analysis

Data were presented as means ± SEM. Statistical analyses were conducted using the StatView 5 system (Statview, Berkeley, CA). The differences between smokers and non-smokers were analyzed by Student's t-test. When more than two groups were compared, the significance of differences between any two groups was determined using Bonferroni's multiple range test. *p *< 0.05 was considered significant.

## Results

Table 1 shows the clinical profiles in the subjects studied. They were all Japanese males with normal glucose tolerance. They were divided into two groups based on the questionnaire for smoking; one with current smokers (n = 48) and the other with current non-smokers (n = 94). There was no significant difference in age (51.5 ± 1.2 vs 52.5 ± 1.0 yr, *p *= 0.286), BMI (23.1 ± 0.4 vs 23.2 ± 0.2 kg/m^2^, *p *= 0.389), waist circumference (84.5 ± 1.0 vs 83.8 ± 0.7 cm, *p *= 0.286), body fat percentage (21.2 ± 0.7 vs 20.6 ± 0.4%, *p *= 0.217), systolic (119 ± 2 vs 122 ± 1 mmHg, *p *= 0.077) and diastolic (75 ± 1 vs 75 ± 1 mmHg, *p *= 0.481) blood pressure, fasting glucose (95 ± 1 vs 95 ± 1 mmHg, *p *= 0.473), HbA1c (5.12 ± 0.04 vs 5.15 ± 0.03%, *p *= 0.250), fasting insulin (3.8 ± 0.3 vs 3.9 ± 0.2 μU/ml, *p *= 0.408), HOMA-IR (0.88 ± 0.08 vs 0.90 ± 0.05, *p *= 0.403), total cholesterol (199 ± 5 vs 204 ± 3 mg/dl, *p *= 0.190), triglyceride (130 ± 11 vs 121 ± 4 mg/dl, *p *= 0.328), and LDL cholesterol (116 ± 5 vs 117 ± 3 mg/dl, *p *= 0.437) between the two groups. Although 2 hour glucose concentration after 75 g oral glucose load was similar between smokers and non-smokers (115 ± 2 vs 112 ± 2 mg/dl, *p *= 0.158), HDL cholesterol was significantly lower in smokers than in non-smokers (55 ± 2 vs 60 ± 1 mg/dl, *p *= 0.031). Whereas TNF-α(1.64 ± 0.15 vs 1.47 ± 0.09 pg/ml, *p *= 0.161) and sTNF-R2(1997 ± 58 vs 1901 ± 45 pg/ml, *p *= 0.102) were not significantly different between the two groups, sTNF-R1 (1203 ± 30 vs 1116 ± 22 pg/ml, *p *= 0.010) and WBC counts (7165 ± 242 vs 5590 ± 168/μl, *p *< 0.001) were significantly higher in smokers than in non-smokers. No significant difference was observed in HMW adiponectin (5.6 ± 0.6 vs 5.4 ± 0.4 μg/ml, *p *= 0.358) between the two groups.

**Table 1:** Clinical characteristics of the 142 male subjects studied.

age (yr)	52.1 ± 0.8
body mass index (kg/m^2^)	23.2 ± 0.2

waist circumference (cm)	84.0 ± 0.6

body fat percentage (%)	20.8 ± 0.4

systolic blood pressure (mmHg)	121 ± 1

diastolic blood pressure (mmHg)	75 ± 1

fasting glucose (mg/dl)	95 ± 1

2 hr glucose after OGTT (mg/dl)	113 ± 5

HbA1c (%)	5.14 ± 0.02

total cholesterol (mg/dl)	202 ± 3

triglyceride (mg/dl)	124 ± 10

HDL cholesterol (mg/dl)	59 ± 1

LDL cholesterol (mg/dl)	117 ± 3

serum creatinine (mg/dl)	0.80 ± 0.01

insulin (μU/ml)	3.82 ± 0.18

HOMA-IR	0.90 ± 0.18

TNF-α (pg/ml)	1.53 ± 0.08

sTNF-R1 (pg/ml)	1145 ± 180

sTNF-R2 (pg/ml)	1933 ± 356

HMW adiponectin (μg/ml)	5.5 ± 0.3

WBC (/μl)	6123 ± 151

Values represent mean ± SEM.

Our present study showed that there was a wide variation in Brinkman index in 48 current smokers (range, 100 to 1840). We therefore classified 48 current smokers into two groups based on the value of Brinkman index: one with heavy smoking (Brinkman index ≧ 600) and the other with light smoking (Brinkman index < 600) (Table 2).

**Table 2:** Clinical characteristics in non-smokers and smokers.

	non-smokers	smokers	
		**light smoking**	**heavy smoking**

number of subjects	94	24	24

Brinkman index	0	385 ± 27^A^	914 ± 59^AB^

number of tobacco smoked per day	0	15.2 ± 0.9^A^	28.3 ± 1.4^AB^

inhaled year (yr)	0	25.8 ± 1.4^A^	32.6 ± 1.4^AB^

age (yr)	52.5 ± 1.0	49.3 ± 1.7	53.8 ± 1.5

body mass index (kg/m^2^)	23.2 ± 0.2	22.8 ± 0.6	23.3 ± 0.5

body fat percentage (%)	20.6 ± 0.4	20.8 ± 1.0	21.6 ± 1.0

waist circumference (cm)	83.8 ± 0.7	84.1 ± 1.4	84.9 ± 1.5

systolic blood pressure (mmHg)	122 ± 1	120 ± 3	117 ± 2

diastolic blood pressure (mmHg)	75 ± 1	76 ± 2	74 ± 2

fasting glucose (mg/dl)	95 ± 1	97 ± 1	92 ± 1

2 hr glucose after OGTT (mg/dl)	112 ± 2	115 ± 4	116 ± 3

HbA1c (%)	5.15 ± 0.03	5.10 ± 0.05	5.14 ± 0.06

total cholesterol (mg/dl)	204 ± 3	193 ± 7	205 ± 7

triglyceride (mg/dl)	121 ± 14	138 ± 18	123 ± 13

HDL cholesterol (mg/dl)	60 ± 1	57 ± 3	54 ± 3

LDL cholesterol (mg/dl)	117 ± 3	109 ± 7	124 ± 7

serum creatinine (mg/dl)	0.81 ± 0.01	0.81 ± 0.01	0.79 ± 0.02

insulin (μU/ml)	3.8 ± 0.2	3.5 ± 0.4	4.1 ± 0.6

HOMA-IR	0.90 ± 0.05	0.83 ± 0.09	0.93 ± 0.13

TNF-a (pg/ml)	1.47 ± 0.09	1.82 ± 0.28	1.46 ± 0.11

sTNF-R1 (pg/ml)	1116 ± 21	1099 ± 30	1307 ± 44^AB^

sTNF-R2 (pg/ml)	1901 ± 44	1827 ± 62	2166 ± 86^AB^

HMW adiponectin (μg/ml)	10.7 ± 0.8	11.4 ± 1.7	11.1 ± 1.5

leptin (ng/ml)	25.6 ± 1.4	23.2 ± 2.0	24.5 ± 2.4

WBC (/μl)	5590 ± 163	6829 ± 352^A^	7500 ± 324^A^

(light smoking & heavy smoking) Values represent mean ± SEM.

^A^: *p*< 0.01 (vs. non-smokers) ^B^: *p*< 0.01 (vs. light smoking)

The subpopulations did not differ with respect to age, BMI, waist circumference, body fat percentage, systolic and diastolic blood pressure, fasting glucose, HbA1c, serum creatinine, lipid profile, HOMA-IR, and HMW adiponectin. Whereas no significant difference was observed in WBC counts and TNF-α, sTNF-R1 and sTNF-R2 were significantly higher in heavy smoking group than in light smoking group, whose sTNF-R1 and sTNF-R2 were similar to those of non-smokers. In contrast, WBC counts were significantly higher in heavy or light smoking group as compared to non-smokers. There was, however, no significant difference in WBC counts between the two smoking groups.

Finally, we compared WBC counts and TNF system activities taking into account the inhaled year and the number of tobacco smoked. Inhaled year did not significantly affect WBC counts or TNF system activities in smokers (data not shown). In contrast, the number of tobacco smoked differently affected WBC and TNF in smokers (Table 3). Irrespective of the number of tobacco smoked, WBC counts were significantly higher in smokers as compared to non-smokers. However, no significant difference was observed in WBC counts between smokers who smoked < 20 cigarettes per day and those who smoked ≧ 20 cigarettes per day. On the other hand, TNF system activities (sTNF-R1, sTNF-R2) were not statistically significant between non-smokers and current smokers who smoked < 20 cigarettes per day, but they were significantly higher in current smokers who smoked ≧ 20 cigarettes per day as compared to those who smoked < 20 cigarettes per day or to non-smokers.

**Table 3:** Clinical characteristics in non-smokers and smokers

	non-smokers	smokers	
		**light smoking**	**heavy smoking**

number of subjects	94	24	24

number of cigarettes a day	0	12.3 ± 0.8^A^	26.1 ± 1.2^AB^

inhaled year (yr)	0	27.8 ± 2.0^A^	29.8 ± 1.3^A^

Brinkman index	0	348 ± 37^A^	786 ± 59^AB^

age (yr)	52.5 ± 1.0	52.5 ± 1.9	51.1 ± 1.5

body mass index (kg/m^2^)	23.2 ± 0.2	22.2 ± 0.7	23.5 ± 04

waist circumference (cm)	83.8 ± 0.7	82.6 ± 1.7	85.3 ± 1.3

body fat percentage (%)	20.6 ± 0.4	19.7 ± 1.4	21.9 ± 0.7

systolic blood pressure (mmHg)	122 ± 1	120 ± 3	118 ± 2

diastolic blood pressure (mmHg)	75 ± 1	75 ± 2	75 ± 2

fasting glucose (mg/dl)	95 ± 1	95 ± 2	94 ± 1

2 hr glucose after OGTT (mg/dl)	112 ± 2	114 ± 5	116 ± 3

HbA1c (%)	5.15 ± 0.03	5.09 ± 0.07	5.14 ± 0.04

total cholesterol (mg/dl)	204 ± 3	198 ± 8	200 ± 6

triglyceride (mg/dl)	121 ± 14	140 ± 20	126 ± 13

HDL cholesterol (mg/dl)	60 ± 1	55 ± 4	56 ± 3

LDL cholesterol (mg/dl)	117 ± 3	116 ± 7	117 ± 7

serum creatinine (mg/dl)	0.81 ± 0.01	0.78 ± 0.04	0.78 ± 0.02

insulin (μU/ml)	3.9 ± 0.2	3.2 ± 0.5	4.0 ± 0.4

HOMA-IR	0.90 ± 0.05	0.77 ± 0.13	0.93 ± 0.10

TNF-a (pg/ml)	1.47 ± 0.09	2.00 ± 0.45	1.47 ± 0.09

sTNF-R1 (pg/ml)	1116 ± 22	1072 ± 37	1263 ± 36^AB^

sTNF-R2 (pg/ml)	1901 ± 45	1767 ± 80	2101 ± 70^ab^

HMW adiponectin (μg/ml)	10.7 ± 0.8	12.8 ± 2.2	10.5 ± 1.3

leptin (ng/ml)	25.6 ± 1.4	20.9 ± 2.3	25.2 ± 2.0

WBC(/μl)	5590 ± 168	6687 ± 396^a^	7382 ± 298^A^

(light smoking & heavy smoking) Values represent mean ± SEM.

^A^: *p *< 0.01 (vs. non-smokers) ^B^: *p *< 0.01 (vs. light smoking)

^a^: *p *< 0.05 (vs. non-smokers) ^b^: *p *< 0.05 (vs. light smoking)

## Discussion

In the present study, we confirmed that not only white blood cell (WBC) counts but also tumor necrosis factor (TNF) system activities are increased in current smokers when compared with current non-smokers. However, smoking affected differently on WBC counts and TNF system activities. Light smoking was associated with WBC counts, while heavy smoking was associated with TNF system activities.

As an index of active smoking, we used Brinckman index in the current study. Brinkman index is calculated as the product of the number of tobacco smoked and the inhaled year. Thus, it is not clear whether which of the two variables is responsible for the increase in WBC counts or the activation in TNF system in smokers. We therefore compared WBC counts and TNF system activities taking into account the inhaled year and the number of tobacco smoked. Inhaled year did not significantly affect WBC counts or TNF system activities in smokers. In contrast, the number of tobacco smoked differently affected WBC and TNF system in smokers (Table 3). Thus, the number of tobacco smoked per se seems to be independently responsible for the increase in WBC counts and TNF activation in our current smokers.

The reason why heavy smoking is associated with TNF system activity is not known, but it may suggest that the smoke-induced activation of TNF system usually requires a significant amount of smoke exposure. This idea is supported from the observation shown by Zoppini et al. [16] that a marked increase of TNF-α system activation was observed with an increase in the number of cigarettes smoked.

Of particular note in the present study is that these findings were obtained after taking into account some variables including BMI, waist circumference, body fat percentage, blood glucose, HbA1c, and HMW adiponectin which are considered to affect TNF system activities and/or WBC counts [17,18]. It may be argued that smoking is associated with visceral fat accumulation. It seems, however, unlikely since Komiya et al. [19] did not fail to draw a conclusion that smoking is a strong and an independent risk factor for inducing excessive accumulation of visceral fat to a greater extent than sedentary life style or heavy alcohol consumption in Japanese men.

The mechanisms by which smokers have an increase in sTNF-R1 are not known at present. The possible explanation is that smoking exerts an inflammatory stimulus on macrophage which causes the release of TNF-α. One of the major organs contributing to the production of TNF may be the pulmonary alveolar cells. Another organ might be periodontal tissue. It is reported that TNF-α content of the gingival crevicular fluid from smokers is reported to be significantly increased as compared with non-smokers [20]. Whereas we did not check the status of periodontal disease in the subjects studied, we previously demonstrated that sTNF-R1 serum levels were independently associated with the severity of periodontal disease in Japanese subjects [21]. In addition, our team previously reported that the therapy of periodontal disease leads to the reduction in TNF-α in Japanese subjects [22].

It may be argued that adipose tissue might be another organ responsible for the increased TNF system activity in smokers. It seems, however, unlikely, since mean BMI in our subjects was 23.2 kg/m^2^. In vitro study shown by Kern et al. [23] disclosed that adipose tissue in the subjects with a BMI greater than 30 kg/m^2 ^released more TNF than those with a BMI less than 25 kg/m^2^.

Whereas the contributory factors underlying tobacco-induced atherosclerosis in humans remain to be elucidated, our present study might suggest that sTNF-R1 could become one of the candidates responsible for tobacco-induced atherosclerosis in humans. Rauchhaus et al. [24] disclosed that sTNF-R1 is predictive of cardiovascular mortality in patients with chronic heart failure. Our team demonstrated that sTNF-R1 is associated with albuminuria or serum homocysteine in Japanese type 2 diabetic patients [10,25]. Not only albuminuria but also serum homocysteine are known to be responsible for the development of atherosclerosis in man. Pinto-Sietsma et al. reported that smoking is related to albuminuria in humans [26]. Jatoi et al. [27] showed a significant linear relationship between smoking status and pulse wave velocity in man. We previously found that TNF system activities are independently associated with pulse wave velocity in non-obese Japanese type 2 diabetic patients [12].

## Conclusions

In summary, it can be concluded that smoking is associated with increased WBC counts and increased activity of the TNF system, but the effect of smoking on WBC counts and TNF system activity was different in Japanese male subjects with normal glucose tolerance. Light smoking was associated with an increase in WBC counts, while heavy smoking was responsible for TNF activation in Japanese male subjects with normal glucose tolerance.

## Abbreviations

WBC: White Blood cell; TNF: tumor necrosis factor; BMI: body mass index; HMW: high molecular weight; sTNF-R: soluble TNF receptor; HOMA-IR: homeostasis model assessment-insulin resistance

## Competing interests

The authors declare that they have no competing interests.

## Authors' contributions

NW researched data, wrote the manuscript and contributed to discussion, MF wrote the manuscript, contributed to discussion and reviewed the manuscript, AT wrote the manuscript, contributed to discussion and reviewed the manuscript, TO contributed to discussion, YN contributed to discussion, FN contributed to discussion, SA contributed to discussion, YI contributed to discussion, RO contributed to discussion, DY contributed to discussion, YN contributed to discussion and reviewed the manuscript, SN contributed to discussion and reviewed the manuscript. All authors read and approved the final manuscript.
